# Comparative analysis of carbapenemases, RND family efflux pumps and biofilm formation potential among *Acinetobacter baumannii* strains with different carbapenem susceptibility

**DOI:** 10.1186/s12879-021-06529-2

**Published:** 2021-08-20

**Authors:** Yanpeng Zhang, Bing Fan, Yong Luo, Zhiyuan Tao, Yongbo Nie, Yongtao Wang, Fanglin Ding, Yanwu Li, Dayong Gu

**Affiliations:** 1 Department of Clinical Laboratory, Shenzhen Institute of Translational Medicine, The First Affiliated Hospital of Shenzhen University, Shenzhen Second People’s Hospital, No. 3002, Sungang Xi Road, Shenzhen, 518035 China; 2 Department of Clinical Laboratory, Wuhan No.1 Hospital, Zhongshan Road, Wuhan, China

**Keywords:** *Acinetobacter baumannii*, Carbapenemases, Efflux pump, Biofilm, Resistance

## Abstract

**Aim:**

This study has conducted a comparative analysis of common carbapenemases harboring, the expression of resistance-nodulation-cell division (RND) family efflux pumps, and biofilm formation potential associated with carbapenem resistance among *Acinetobacter baumannii (A. baumannii*) strains with different carbapenem susceptibility. Methods: A total of 90 isolates of *A. baumannii* from two tertiary hospitals of China were identified and grouped as carbapenem susceptible *A. baumannii* (CSAB) strains and carbapenem non-susceptible *A. baumannii* (CnSAB) strains based on the susceptibility to imipenem. Harboring of carbapenemase genes, relative expression of RND family efflux pumps and biofilm formation potential were compared between the two groups. Result: Among these strains, 12 (13.3 %) strains were divided into the CSAB group, and 78 (86.7 %) strains into the CnSAB group. Compared with CSAB strains, CnSAB strains increased distribution of *bla*_OXA−23_ (*p* < 0.001) and IS*Aba1*/*bla*_OXA−51−like_ (*p* = 0.034) carbapenemase genes, and a 6.1-fold relative expression of *adeB* (*p =* 0.002), while CSAB strains led to biofilm formation by 1.3-fold than CnSAB strains (*p* = 0.021).

**Conclusions:**

Clinically, harboring more *bla*_OXA−23−like_ and IS*Aba1/bla*_OXA−51−like_ complex genes and overproduction of adeABC are relevant with carbapenem resistance, while carbapenem susceptible strains might survive the stress of antibiotic through their ability of higher biofilm formation.

## Introduction

*Acinetobacter baumannii* (*A. baumannii*) is emerging as an opportunistic nosocomial pathogen and clinically causes serious infections, including ventilator-associated pneumonia, urinary tract infection, surgical wound infection, pyemia, meningitis, and peritonitis [1–4]. *A. baumannii* has become a clinically successful pathogen owing to its strong tolerance to an adverse environment, complex drug resistance mechanism and plastic genome. This pathogen has caused serious public health problems globally in recent 20 years [5].

Carbapenems, including imipenem, meropenem and doripenem, are important antibiotics in treating *A. baumannii* infections [6, 7]. According to the results of the antimicrobial sensitive test (AST), clinical isolates of *A. baumannii* can be divided into carbapenem non-susceptible *A. baumannii* (CnSAB) and carbapenem susceptible *A. baumannii* (CSAB). Even following the guidance of AST strictly, the actual treatment effects of *A. baumannii* are often not so ideal, both for CnSAB and CSAB. Different strains seem to live in different ways. Carrying carbapenemases, overproduction of efflux pumps, low-expression of outer membrane proteins, and biofilm formation may be related to the survival of *A. baumannii* in the presence of antibiotics. A comparative understanding of CSAB and CnSAB pathogen characteristics is critical for adopting appropriate strategies to control their infection. To the best of our knowledge, most studies focused on the resistance mechanisms, and only a few investigations have attempted comparing CnSAB and CSAB, especially after modifying the clinical breakpoints for imipenem in 2021 (CLSI 2021, 31st Edition; Document M100). In this context, this study aims to investigate the different characterization in carbapenemases harboring, efflux pumps expression level and biofilm formation capability of CnSAB and CSAB strains of *A. baumannii*.

## Methods

### Collection and identification of strains

A total of 115 non-repetitive strains of *Acinetobacter calcoaceticus - Acinetobacter baumannii* complex were collected from two hospitals from July 2018 to December 2019. 90 of these strains were further identified as *A. baumannii* using *rpoB* gene sequence analysis with a previously described method [8, 9], and the primer sequences are listed in Table 1.

Among them, 48 strains were from the First Affiliated Hospital of Shenzhen University in South China, and 42 strains were from Wuhan No.1 Hospital in Central China. Specimen sources of these isolates included sputum, blood, nasal secretion, alveolar lavage fluid, urine, etc. This study was approved by the Ethics Committee of the First Affiliated Hospital of Shenzhen University (approval ID 20200511007). The data related to this study were received from the hospital’s information system, and the patient’s informed consent was exempted. Overall, the outcome of this study is expected to benefit patients with the infection of *A. baumannii* for better therapy.

### Antimicrobial susceptibility test (AST)

AST of the above strains was determined by an automated broth microdilution method (Gram-negative susceptibility cards) through the VITEK 2 system (Biomerieux, France) according to the manufacture’s instruction, and susceptibility interpretation was based on the clinical breakpoints from the Clinical and Laboratory Standards Institute (CLSI 2021, 31st Edition; Document M100). *Escherichia coli* ATCC 25,922 and *Pseudomonas aeruginosa* ATCC 27,853 strains were used as the control. According to the CLSI clinical breakpoint to imipenem, these strains were divided into two groups: carbapenem susceptible *A. baumannii* (CSAB) and carbapenem non-susceptible *A. baumannii* (CnSAB). In CSAB, the minimum inhibitory concentration (MIC) for imipenem was ≤ 2 µg/ml; and in CnSAB, MIC was ≥ 4 mg/L (intermediate: 4 mg/L; resistant: ≥8 mg/L).

### Detection of carbapenemases

Based on the already established polymerase chain reaction (PCR) conditions [10], all these 90 strains were subjected to the detection of carbapenemase genes, including *bla*_SIM_ (Seoul imipenemase), *bla*_VIM_ (Verona integron-encoded metallo-β-lactamases), *bla*_IMP_ (imipenemase), *bla*_NDM−1_ (New-Delhi metallo-β-lactamase), *bla*_OXA−51−like_, *bla*_OXA−23−like_, *bla*_OXA−24/40−like_ and *bla*_OXA−58−like_ [11, 12]. The detection of IS*Aba1*/*bla*_OXA−51−like_ was performed using the method of Jane Turton et al [13]. All PCR primers targeting resistance genes and mobile elements used in this study are shown in Table 1.

**Table 1:** Primer sequences of targeting genes and amplicon size

Target genes	Primer sequence	Size (bp)
*rpoB*	F: GAGTCTAATGGCGGTGGTTC R: ATTGCTTCATCTGCTGGTTG	110
*bla*_SIM_	F:TACAAGGGATTCGGCATCG R:TAATGGCCTGTTCCCATGTG	570
*bla*_VIM_	F: GATGGTGTTTGGTCGCATA R: CGAATGCGCAGCACCAG	390
*bla*_IMP_	F: GGAATAGAGTGGCTTAAYTC R: TCGGTTTAAYAAAACAACCACC	232
*bla*_NDM−1_	F: GGTTTGGCGATCTGGTTTTC R: CGGAATGGCTCATCACGATC	621
*bla*_NDM−1_	F: GAGTATTCAACATTTCCGTGTC R: TAATCAGTGAGGCACCTATCTC	850
*bla*_OXA−23−like_	F: GAATATGTGCCAGCCTCTAC R: GCATTACCGAAACCAATACG	245
*bla*_OXA−24/40−like_	F: TGGGTGGAGCAAGCTAATGG R: ACGAATAGAACCAGACATTCCTTCT	81
*bla*_OXA−51−like_	F: TAATGCTTTGATCGGCCTTG R:TGGATTGCACTTCATCTTGG	353
*bla*_OXA−58−like_	F: GACAATTACACCTATACAAGAAG R: AAACCCACATACCAACCC	599
IS*Aba1/bla*_OXA−51_	F: CACGAATGCAGAAGTTG R: CTTCTGTGGTGGTTGGC	1200
*adeB*	F: GCAGAGCGTACTCGGAATGT R: CCACTGAAACCCCATCCCAA	101
*adeG*	F: GGTGAATTACTTGGTGATGC R: TTTGGTCAGGCGCAGGTATT	86
*adeJ*	F: TTCGGTGGCTCATACGCAAT R: GGAGCACCACCTAACTGACC	137
*16 S rRNA*	F:AGCTAACGCGATAAGTAGACCG R: TGTCAAGGCCAGGTAAGGTTC	137

### Relative expression of RND family efflux pumps

The relative gene expressions of *adeB, adeJ* and *adeG* were used to evaluate the relative expressions of adeABC, adeFGH and adeIJK efflux pumps, respectively. The preparation methods of RNA templates and quantitative real-time PCR assays were performed based on the earlier reported conditions [10]. The 16 S rRNA gene as control and *A. baumannii* ATCC 17,978 as a reference strain were used to measure the relative expression levels. All the reactions were carried out in triplicate.

### Detection of biofilm production capacity

The biofilm formation assay was performed according to the previous method [14]. *A. baumannii* strains were cultured overnight and diluted to a density of 0.5 on the McFarland scale. 100 µl of the diluted culture was introduced into the wells of 96-well plate and incubated for 24 h at 37 °C without shaking. All wells were washed three times, and the planktonic bacteria were removed. 125 µl (0.1 %) crystal violet solution was then added into each well and incubated for 10 min at 25 °C. The wells were then washed and dried at room temperature. Each well was then added 200 µl of 95 % ethanol and incubated for 10 min at 25 °C. The obtained ethanol-crystal violet solution in each well was transferred to a new 96-well plate to determine the optical density (OD) at 550 nm.

### Statistical analysis

Data entry and analysis were performed with Stata/SE 15.1 for windows version 16.0 (Stata Corp LLC, Texas, USA). Categorical variables were described as frequency numbers (percentages). The distribution of sources of samples, age and gender, were compared using Pearson’s chi-square test. Harboring of resistance genes, relative expression of efflux pumps, and biofilm formation between the CSAB and CnSAB groups were compared using the Fisher-Exact or independent *t*-test. The association of relative expression of RND family efflux pumps and biofilm formation were analyzed using multivariate regression. All tests were two-tailed, and a *p <* 0.05 was considered statistically significant.

## Results

### Clinical characteristic of strains

The clinical characteristics of the 90 isolates of *A. baumannii* are listed in Table 2. In this study, the patients who suffered from the infection of *A. baumannii* consisted of 72 (80.0 %) males and 18 (20.0 %) females. Among them, 52 (57.8 %) were over 60 years old (including 60), and 38 (42.2 %) were less than 60 years old. Respiratory tract specimens were the most frequent source. These respiratory tract specimens included sputum, bronchoalveolar lavage fluid and nasal secretion, and their respective proportions were 71.1 %, 8.9 and 5.6 %, respectively. Five of these strains were isolated from a blood specimen.

**Table 2:** Characteristics of 90 isolates of *A. baumannii*

Variables	CSAB	CnSAB	*P*-value	Number
Age			0.22	
< 60	7 (58.3 %)	31 (39.7 %)		38 (42.2 %)
≥ 60	5 (41.7 %)	47 (60.3 %)		52 (57.8 %)
Gender			0.73	
Female	2 (16.7 %)	16 (21 %)		18 (20.0 %)
Male	10 (83.3 %)	62 (79 %)		72 (80.0 %)
Specimen types			0.28	
Sputum	8 (66.7 %)	56 (71.8 %)		64 (71.1 %)
Urine	1 (8.3 %)	4 (5.1 %)		5 (5.6 %)
Blood	2 (16.7 %)	3 (3.8 %)		5 (5.6 %)
Bronchoalveolar lavage fluid	0 (0.0 %)	8 (10.3 %)		8 (8.9 %)
Nasal secretion	1 (8.3 %)	4 (5.1 %)		5 (5.6 %)
Skin and soft tissue	0 (0.0 %)	3 (3.8 %)		3 (3.3 %)

### Antimicrobial susceptibility test

Based on the susceptibility to imipenem, strains were divided into CnSAB and CSAB. Of the 90 strains, 13.3 % (12/90) were carbapenem susceptible (CSAB), and the rest (86.7 %, 78/90) were classified as CnSAB.

### Distribution of carbapenemases

As shown in Table 3, no *bla*_SIM_, *bla*_VIM_ and *bla*_IMP_ genes were detected in these 90 isolates. An intrinsic carbapenemase gene to *A. baumannii* species, *bla*_OXA−51−like_ was present in all the 90 (100 %) isolates. The *bla*_OXA−23−like_, *bla*_OXA−24/40−like_ and *bla*_OXA−58−like_ were present in 83.3 % (75/90), 1.1 % (1/90), and 2.2 % (2/90) isolates, respectively. The *bla*_OXA−23−like_ was detected positive in 33.3 % (4/12) of the CSAB group and 91.0 % (71/78) of CnSAB, respectively. Compared with CSAB strains, CnSAB showed a statistically significant increasing distribution of *bla*_OXA−23−like_ (*p <* 0.001). For another carbapenemase gene, IS*Aba1/bla*_OXA−51−like_ was detected in 28.2 % (22/78) in CnSAB group (*p* = 0.034). The *bla*_OXA−24/40−like_ and *bla*_OXA−58−like_ genes were noted in 1 and 2 strains, respectively. *bla*_NDM−1_ gene was detected in 6.4 % (5/78) of CnSAB strains.

**Table 3:** Distribution of Carbapenemase in 90 *A. baumannii*

Gene	CSAB (n = 12)		CnSAB (n = 78)		p	Total (N/%)
	N	Prevalence rate(%)	N	Prevalence rate (%)		
*bla*_SIM_	0	0	0	0	–	0 (0.0)
*bla*_VIM_	0	0	0	0	–	0 (0.0)
*bla*_IMP_	0	0	0	0	–	0 (0.0)
*bla*_NDM−1_	0	0	5	6.4	0.37	5 (5.6)
*bla*_OXA−23−like_	4	33.3	71	91	< 0.001	75 (83.3)
*bla*_OXA−24/40−like_	0	0	1	1.3	0.69	1 (1.1)
*bla*_OXA−51−like_	12	100	78	100	–	90 (100)
*bla*_OXA−58−like_	0	0	2	2.6	0.57	2 (2.2)
IS*Aba1*/*bla*_OXA−51−like_	0	0	22	28.2	0.034	22 (24.4)
*bla*_OXA−23−like_ & *bla*_OXA−24/40−like_	0	0	1	1.3	–	1 (1.1)
*bla*_OXA−23−like_ & *bla*_OXA−58−like_	0	0	2	2.6	–	2 (2.2)
*bla*_OXA−23−like_& IS*Aba1*/*bla*_OXA−51−like_	0	0	22	28.2	0.034	22 (24.4)

### Relative expression of RND family pumps

**Fig. 1:**
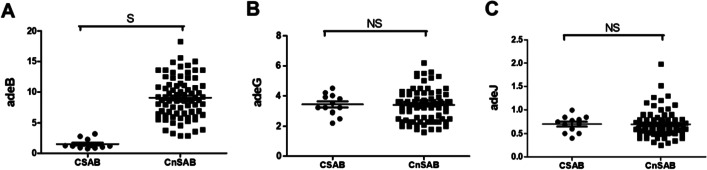
Relative expressions of *adeB, adeJ* and *adeG* in CSAB and CnSAB strains. The RND family efflux pump expression was determined by the quantitative real-time PCR, and *Acinetobacter baumannii* strain ATCC 17,978 was taken as a reference. All reactions were carried out in triplicate. The black dot indicates carbapenem susceptible *Acinetobacter baumannii* (CSAB) strains; the Black square denotes carbapenem non-susceptible *Acinetobacter baumannii* (CnSAB) strains. S, significant; NS, not significant

The relative expression of three RND family efflux pumps genes was measured using quantitative real-time PCR (qRT-PCR) (Fig. 1). Compared with CSAB, the relative expressions of *adeB*, adeJ, and *adeG* genes in CnSAB were increased by 6.1, 0.9, and 0.9 times, respectively. The relative expression of *adeB* was significantly increased (*p* = 0.003), but the relative expressions of *adeG* (*p* = 0.709) and *adeJ* (*p* = 0.340) were not significantly increased.

### Biofilm forming capacity

**Fig. 2:**
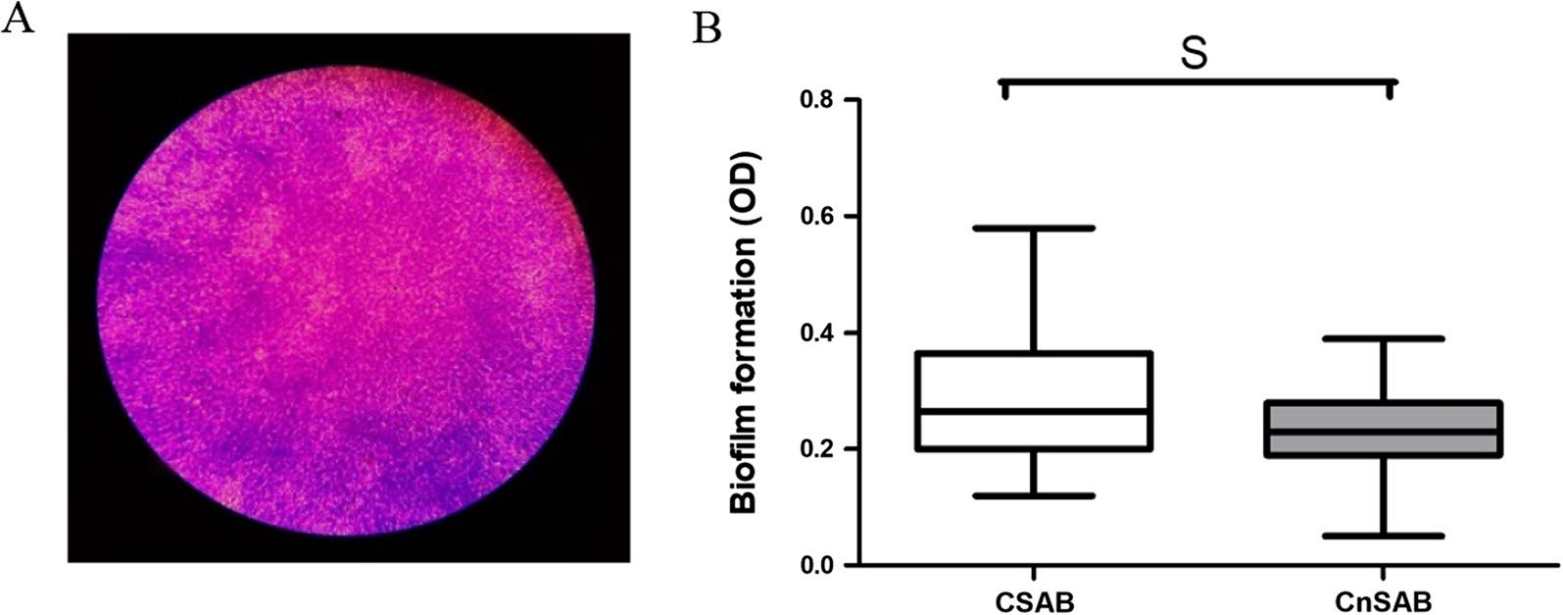
Biofilm formation capacity of CSAB and CnSAB strains. **A** The biofilm of *A. baumannii* by violet crystalline dying (under oil lens, 1000x). **B** Comparison of biofilm production between carbapenem susceptible (CSAB) and carbapenem non-susceptible (CnSAB) groups of *A. baumannii*. S, significant; NS, not significant

The biofilm-forming potential of strains was measured through violet crystalline dying. As shown in Fig. 2, CSAB strains produced biofilm with an OD of 0.29 ± 0.04, while CnSAB strains produced biofilm with an OD of 0.23 ± 0.02. the biofilm produced by CSAB strains was 1.3 times higher than that of CnSAB. There was significant difference in biofilm forming capacity between the two groups (*p* = 0.021).

### Association between the relative expression of RND family efflux pumps and biofilm formation

The regression analysis results of the correlation between the relative expression of RND family efflux pumps and biofilm formation are shown in Table 4. The relative expressions of three proteins, *adeB, adeG, adeJ*, demonstrate no significant association with biofilm formation capacity (*p* = 0.128, 0.218, and 0.601, respectively).

**Table 4:** Association between the relative expression of RND family efflux pumps and biofilm formation

Biofilm	Coef.	Std. err.	*p*	95 % Conf. interval
*adeB*	− 0.004	0.002	0.128	− 0.008 to 0.001
*adeG*	− 0.010	0.008	0.218	− 0.026 to 0.006
*adeJ*	− 0.024	0.047	0.601	0.117 to 0.068

## Discussion

*A. baumannii* is an important pathogen of nosocomial infections. This decade witnessed a series of clinical infections events and led to high clinical mortality [15–19]. Outstanding drug resistance and tolerance to the harsh environment make *A. baumannii* to be a successful clinical pathogen [1, 20]. Earlier studies have observed that powerful resistance was associated with hydrolyzing enzymes, efflux pumps, biofilm and outer membrane in *A. baumannii* [11, 21, 22]. This study was designed to compare the characterization of carbapenemases harboring, efflux pumps relative expression and biofilm formation capability in the *A. baumannii* strains with different carbapenem susceptibility. For this, 90 isolates of *A. baumannii* obtained from two hospitals were identified and were divided into CSAB and CnSAB groups based on their imipenem susceptibility. The obtained results indicate that CnSAB strains increased distribution of *bla*_OXA−23_ (*P* < 0.001) and IS*Aba1*/*bla*_OXA−51−like_ (*p* = 0.034) carbapenemase genes, and a 6.1-fold relative expression of *adeB* (*p =* 0.002) was noted compared with CSAB strains, while the biofilm produced by CSAB strains was 1.3 times higher than that of CnSAB, (*p* = 0.021).

Production of multiple carbapenemases is effective for carbapenem resistance. The most important carbapenemases in *A. baumannii* include class B metallo-β -lactamases (MBLs) and class D oxacillinases (OXAs). OXAs include *bla*_OXA−51−like_, *bla*_OXA−23−like_, *bla*_OXA−24/40−like_ *and bla*_OXA−58−like_, and MBLs include *blaSIM* (Seoul imipenemase), *blaVIM* (Verona integron-encoded metallo-beta-lactamases), *blaIMP* (imipenemase), *blaNDM-1* (New-Delhimetallo-β-lactamase) [23] [24] [25].

Whereas the composition of different carbapenemases was different in different regions, the most common mechanism for carbapenem resistance was harboring *bla*_OXA−23_ in *A. baumannii*. Janak Koirala et al. reported *bla*_OXA−23−like_ (52 %) and *bla*_OXA−24/40−like_ (28 %) were the most common genes in CnSAB in central Illinois, the United States. [26]Sunil Kumar et al. found a high prevalence of *bla*_OXA−23−like_ (97.7 %) among the carbapenem-resistant strains followed by *bla*_NDM−1_ (29.1 %) and *bla*_OXA58−like_ (3.5 %) in India [27]. Udomluk Leungtongkam found a pattern of *bla*_OXA−23_ (82.6 %), *bla*_NDM−1_ (9.1 %), *bla*_OXA−24/40−like_ (0.3 %), and *bla*_OXA−58−like_ (6.5 %) in 339 *A. baumannii* in Thailand [24]. In the current study, a pattern of *bla*_OXA−23−like_ (83.3 %), IS*Aba1/bla*_OXA−51−like_ (24.4 %), *bla*_NDM−1_ (5.6 %), *bla*_OXA−24/40−like_ (1.1 %), and *bla*_OXA−58−like_ (2.2 %) was detected in CnSAB. CnSAB strains showed a statistically significant increase of harboring of *bla*_OXA−23_ (*p* < 0.001) compared with CSAB strains.

Another interesting finding was that harboring the IS*Aba1/bla*_OXA−51−like_ gene might be another important factor in carbapenem resistance. The chromosome encoded *bla*_OXA−51−like_ gene is intrinsic, but the solitary OXA-51 enzyme only showed a weak hydrolyzing activity to carbapenems. While with an additional genetic element IS*Aba1* upstream insertion, the IS*Aba1/bla*_OXA−51−like_ complex may result in OXA-51 overproduction which confers resistance to carbapenem. In this study, a significant difference in the carrying of IS*Aba1/bla*_OXA−51−like_ between CnSAB and CSAB strains (*p* = 0.034) was observed. In this study, four isolates harboring *bla*_OXA−23−like_ were found susceptible to carbapenem. This may be due to some mutations in *bla*_OXA−23−like_ gene or decrease of expression of this gene. Based on this, a further quantitative detection for *bla*_OXA−23−like_ expression is necessary to evaluate the actual contribution to carbapenem resistance in further research.

Besides carbapenemase, overproduction of efflux pumps could be another important factor contributing to drug resistance. Efflux pumps can extrude a variety of antimicrobial agents and reduce the accumulation of antibiotics in bacteria. According to the reports in this decade, five super families of efflux pumps have been found in *A. baumannii*: the resistance-nodulation-cell division (RND) family, the ATP-binding cassette (ABC) transporters family, the multidrug and toxic compound extrusion (MATE) family, the major facilitator super (MFS) family, and the small multidrug resistance (SMR) family [10, 28–31]. The three RND efflux family members, adeABC, adeFGH and adeIJK, are the most important pumps for carbapenem resistance in *A. baumannii*. The relative expressions of *adeB, adeJ* and *adeG* were commonly used to evaluate the relative expression of adeABC, adeFGH, and adeIJK efflux pumps, respectively. Also, by comparing with CSAB strains, the relative expression of *adeB*, *adeJ*, and *adeG* genes in CnSAB strains increased by 6.1-fold (*p* = 0.003), 0.9-fold (*p* = 0.709), and 0.9-fold (*p* = 0.340), respectively. These results agree with Yili Chen’s findings, where they found a significantly increased expression of *adeB* from carbapenem-resistant *A. baumannii* strains compared with susceptible strains [32]. This indicates the overproduction of adeABC efflux pump might be another potential cause for carbapenem resistance. In this study, only RND family pumps were discussed, but the contribution of other efflux family pumps needs to be examined further.

Biofilm formation of *A. baumannii* often leads to relapse of chronic infection and disease delay. The relationship between biofilm formation potential and resistance of carbapenems is controversial. Using confocal laser scanning microscopy, Dahdouh et al. found that carbapenem resistant strains could produce more biofilm than susceptible strains [33]. In comparison, Perez et al. reported an inverse relationship between the biofilm formation ability and carbapenem resistance level. They observed meropenem susceptible isolates produced more biofilm than the resistant ones in nosocomial *A. baumannii* strains [34]. This study noticed that the biofilm produced by CSAB strains was 1.3 times higher than that of CnSAB, and the difference is significant (*p* = 0.021). It was indicated that the carbapenem susceptible strains might take advantage of the biofilm formation to survive the pressure of antimicrobials. This explains why the clinical therapy for *A. baumannii* sensitive strains is often not so ideal, even following the guide of AST strictly *in vitro*.

The relative expression of RND efflux pumps influencing biofilm formation is controversial. Yoon et al. reported that mutants with up-regulation expression of the AdeABC, AdeFGH and AdeIJK efflux pumps reduced biofilm formation compared with wild strains. In contrast, He et al. reported that overexpression of the AdeFGH efflux pump is beneficial for biofilm formation in *A. baumannii.* In this study, a regression analysis was attempted. No relationship between the relative expressions of adeB, adeG, and adeJ, as well as biofilm formation capacity in these 90 isolates, was found out.

Besides the above observations, this study has several limitations. Firstly, the sample size was relatively small, and the source of the strains was only from two hospitals. Secondly, this study only discussed the relationship of overproduction of three members of the RND family, but other efflux pump families and outer membrane proteins were not involved. Thus, the experimental data analysis might be influenced by these limitations. Thirdly, different resistance mechanisms may have synergistic or antagonistic effects, and the interactions between those resistance mechanisms have not been considered in this study.

## Data Availability

All data generated or analyzed during this study are included in this published article.
